# The Influence of Deep Cryogenic Treatment (DCT) on the Microstructure Evolution and Mechanical Properties of TC4 Titanium Alloy

**DOI:** 10.3390/ma17184603

**Published:** 2024-09-19

**Authors:** Xuzhi Lan, Yulang Xu, Jingyong Li, Yifeng Gong, Mingxiao Shi

**Affiliations:** 1 Advanced Welding Technology Provincial Key Laboratory, Jiangsu University of Science and Technology, Zhenjiang 212003, China; xuzhi_lan@126.com (X.L.); yulang_xu@163.com (Y.X.); 18260626221@163.com (Y.G.); smx_just@163.com (M.S.); 2 School of Naval Architecture & Intelligent Manufacturing, Jiangsu Maritime Institute, Nanjing 211199, China

**Keywords:** TC4, deep cryogenic treatment (DCT), microstructure evolution, mechanical properties

## Abstract

Deep cryogenic treatment (−196 °C, DCT) is an emerging application that can make significant changes to many materials. In this study, DCT was applied to Ti6Al4V (TC4) titanium alloy, and we delved into an examination of the impact on its microstructural morphologies and mechanical properties. It was observed that DCT has a significant effect on the grain refinement of the TC4 titanium alloy base material. Obvious grain refinement behavior can be observed with 6 h of DCT, and the phenomenon of grain refinement becomes more pronounced with extension of the DCT time. In addition, DCT promotes the transformation of the β phase into the α′ phase in the TC4 titanium alloy base material. XRD analysis further confirmed that DCT leads to the transformation of the β phase into the α′ phase. The element vanadium was detected by scanning electron microscopy, and it was found that the β phase inside the base material had transformed into the α′ phase. It was observed that DCT has a positive influence on the hardness of the TC4 titanium alloy base material. The hardness of the sample treated with 18 h of DCT increased from 331.2 HV_0.5_ to 362.5 HV_0.5_, presenting a 9.5% increase compared to the sample without DCT. Furthermore, it was proven that DCT had little effect on the tensile strength but a significant impact on the plasticity and toughness of the base material. In particular, the elongation and impact toughness of the sample subject to 18 h of DCT represented enhancements of 27.33% and 8.09%, respectively, compared to the raw material without DCT.

## 1. Introduction

Heat treatment is an effective way to tailor material properties to their desired applications. There are many traditional heat treatment procedures for titanium alloys [1,2,3]. The advantages and disadvantages of these procedures are mostly known. In the literature [4,5,6], there are many studies about the effect of traditional heat treatments on titanium alloys, such as annealing and quench aging, on their mechanical properties. However, studies about deep cryogenic treatment (DCT) of titanium alloys are limited. DCT is a relatively new method and is generally applied as an additional heat treatment to parts in their near-final shape [7,8,9,10]. This sub-zero treatment generally takes a long soaking time, which might alter the microstructure and mechanical properties [11,12,13,14,15]. Nowadays, some industries like the aerospace, automotive, and electronic industries use this process in their production lines to improve the mechanical properties and dimensional stability of components.

A lot of research has been conducted on DCT in recent years. Çakir et al. [16] found that the internal structure of materials changed, the grains were refined, and some amount of the β phase transformed into the α phase with the application of cryogenic treatment. Furthermore, the internal stresses in the structure decreased. In addition, the effect of shallow (−80 °C) cryogenic treatment on structure is limited compared with deep (−196 °C) cryogenic treatment.

Li et al. [17] observed that the grains rotate and turn from (110) to the (100) and (101) directions with an increase in the DCT time. TEM images revealed that the dislocation density of the DCT sample increased obviously in comparison to that in a sample without DCT, together with apparent tangling.

In research conducted by Gu et al. [18,19,20], an in-depth analysis was carried out to prove that the improvement in wear resistance can be attributed to the refinement of the grain size and the reduction in the β phases after cryogenic treatment. Furthermore, the formation of a high dislocation density and twins can dissipate a large amount of the energy produced by sliding friction and resist the formation of cracks on the worn surface, which improves the wear resistance of the Ti-6Al-4V alloy.

In terms of the present investigation, there are few reports on the application of DCT to the modification of the TC4 titanium alloy. Therefore, this study delved into an examination of the impact of DCT on the microstructural morphologies and mechanical properties in TC4 titanium alloy, which hopefully will provide reliable experimental references and foundational data for research on DCT modification of TC4 titanium alloy.

## 2. Experimental Procedures

### 2.1. Testing Materials

The base materials chosen for DCT were TC4 titanium alloy plates, measuring 100 mm × 100 mm × 3 mm (length × width × thickness). As is known, TC4 titanium alloy is a biphasic alloy which comprises a composite structure of the α + β phase. Its main alloying elements are Al and V. Al, as an α stabilizing element, enhances the room temperature strength and thermal strength of alloys through solid solution strengthening of the α phase. V, as a solid solution strengthening element, not only enhances the strength of the alloy but also improves its plasticity. Table 1 shows the chemical elements of TC4 titanium alloy, and Table 2 shows the main mechanical properties of TC4 titanium alloy.

The DCT method applied in this experiment was liquid nitrogen immersion, and the DCT temperature was −196 °C. The samples were put into a deep cryogenic tank and then transferred back into the atmosphere until they returned to room temperature. In this experimental context, a selection of five different DCT time was administered, and the DCT times were designed as 0 h, 6 h, 12 h, 18 h, and 24 h separately; then, the corresponding samples were numbered DCT0, DCT6, DCT12, DCT18, and DCT24.

### 2.2. Testing Equipment

A comprehensive array of investigative techniques was applied to unravel the intricate mechanisms underpinning the influence of DCT upon the TC4 titanium alloy. These techniques encompassed optical microscopy (SZ61 and BX51M; Olympus Co., Ltd., Tokyo, Japan), XRD (SmartLab9kW; Rigaku Co., Ltd., Tokyo, Japan), and EDS/EBSD (S3400N; Hitachi Co., Ltd., Tokyo, Japan). The microstructure observation sample for this experiment was set to 10 mm × 3 mm × 3 mm, cut in the rolling direction and polished sequentially with 400 #, 800 #, 1200 #, 1500 #, and 2000 # abrasive paper. After polishing, it was polished with a 0.05 µm SiO_2_ suspension and then using Kroll reagent (V_HNO3_:V_HF_:V_H2O_ = 1:2:18) for corrosion.

Concurrently, mechanical assessments were carried out, including of Vickers hardness (KB30S, KB Co., Ltd., Assenheim, Germany), tensile properties (CMT5205, MTS, Minneapolis, MN, USA), and impact properties (SANS, ZBC2302-D, Metes Industrial Systems Co., Ltd., Shanghai, China). Measurements of hardness were obtained at intervals of 0.2 mm under a load of 500 g for 15 s, and five points for each specimen were tested and averaged as the final results. Tensile tests were performed with a tensile rate of 2 mm/min at room temperature; three specimens were tested for each group, and then their values were averaged as the final values.

## 3. Results and Discussion

### 3.1. Microstructure Evolution Analysis

**Figure 1:**
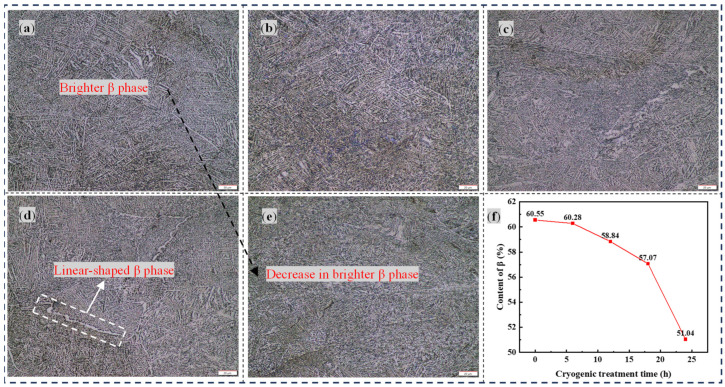
Metallographic structure of the base material treated with different DCT times: (**a**) 0 h, (**b**) 6 h, (**c**) 12 h, (**d**) 18 h, and (**e**) 24 h. (**f**) Percentage content of β.

Figure 1 shows the metallographic structure of the TC4 titanium alloy raw metal treated with different DCT times. From Figure 1a, it can be observed that the raw material of the TC4 titanium alloy is composed of a darker sheet-like α phase and a brighter β phase [21]. Furthermore, it can be seen from Figure 1b that the metallographic structure of the TC4 titanium alloy does not change much with 6 h of DCT, and the interior structure is still composed of the α + β phases. Following the introduction of DCT, it can be clearly seen from Figure 1c that the microstructure has a certain degree of refinement when the DCT time reaches 12 h, showing a decrease in the brighter β phase compared to those treated for 0 h and 6 h. This phenomenon indicates that the β phase inside the TC4 titanium alloy base material may undergo a phase transformation during DCT. It can be seen that the content of the β phase has obviously been changed after DCT, as demonstrated using IPP (Image–Pro Plus) software 1.53a and shown in Figure 1f.

From the analysis of the effects of different DCT times on the metallographic structure of the base material, it is concluded that the structure of the raw material can be refined with DCT. The phenomenon of grain refinement is attributed to the changes in the internal stress of the material. During the cryogenic process, the cryogenic environment causes the material to have a tendency to shrink as a whole. During the shrinkage process, some unstable grains will break down into small grains. Therefore, the phenomenon of grain refinement occurs.

### 3.2. Phase Composition Analysis

#### 3.2.1. XRD Analysis

**Figure 2:**
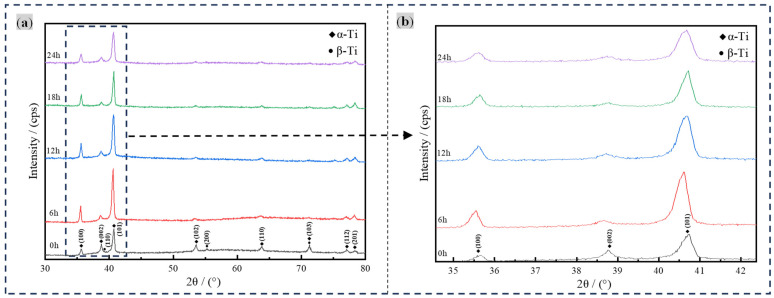
XRD diffraction patterns. (**a**) XRD diffraction patterns of the base material treated with different DCT times, (**b**) Patitial XRD patterns of (**a**).

In terms of the XRD analyses, TC4 titanium alloy subjected to DCT and that without DCT, under otherwise identical conditions, were examined. As shown in Figure 2a,b, the phase composition in the base material remained unaltered, consisting solely of α–Ti and β–Ti [22].

The three strong peaks in the titanium alloy base material are (100), (002), and (101) peaks, as shown in Figure 2b. It can be seen that the (100) and (101) peaks of the three strong peaks were significantly strengthened by DCT. There are two main reasons for this strengthening phenomenon. Firstly, the transformation of the β–Ti phase into the α–Ti phase increases the content of the α phase in the TC4 titanium alloy base material. Secondly, the grains rotate due to the effect of cryogenic internal stress with DCT, and the preferred orientation of the crystal planes is (100) and (101). Meanwhile, the (002) peak shows a certain degree of decrease.

#### 3.2.2. SEM Analysis

**Figure 3:**
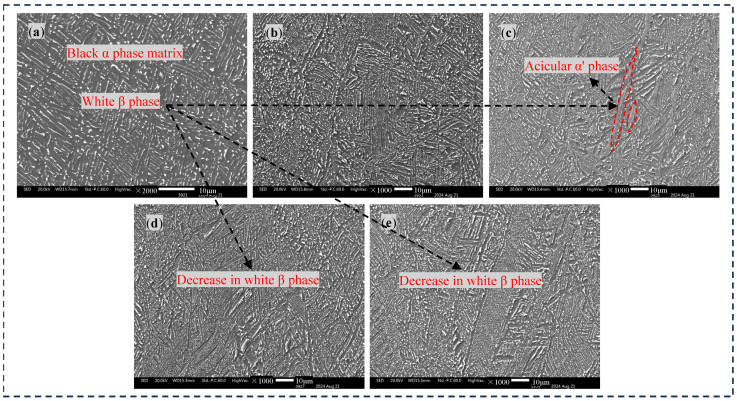
SEM morphologies of the base material treated with different DCT times: (**a**) 0 h, (**b**) 6 h, (**c**) 12 h, (**d**) 18 h, and (**e**) 24 h.

Figure 3 shows the SEM morphologies of the TC4 titanium base material with different DCT times. From Figure 3a, it can be clearly seen that there are a number of white β phases on the black α phase matrix of the raw material, and the β phases are generally connected. From Figure 3b, it can be observed that the originally white β phase became smaller with 6 h of DCT, but the proportion change between the β phase and the α phase was not significant. The β phase was further refined with 12 h of DCT, the content of the white β phase continued to decrease, and precipitation of some of the needle-shaped α′ phase was clearly observed, suggesting the presence of a needle-shaped martensite α′ phase. Based on the XRD test results mentioned earlier, it is inferred that there is a suspected presence of the needle-like martensite α′ phase through the significant increase in diffraction peaks (100) and (101) in the three strong peaks with and without DCT. With the DCT time increased to 18 h or 24 h, the degree of refinement of the β phase is higher, and some of the β phase even becomes small particles, as the cryogenic environment causes the material to have a tendency to shrink as a whole, and during the shrinkage process, some unstable grains will break down into small grains.

In subsequent studies, the presence of needle-shaped martensitic α′ phases was confirmed furtherly by measuring the elemental content. Although needle-shaped martensitic α′ phases have the same dense hexagonal structure as α, their elemental composition is different. When the β phase cools from a high temperature to a low temperature, if the rate is slow enough, the element V can fully precipitate and transform into the α phase. However, when the cooling rate is too fast, the element V cannot precipitate in time, and a supersaturated α phase with the same composition as the β phase element will be formed, which is the needle-like martensite α′ phase.

#### 3.2.3. EDS Analysis

**Figure 4:**
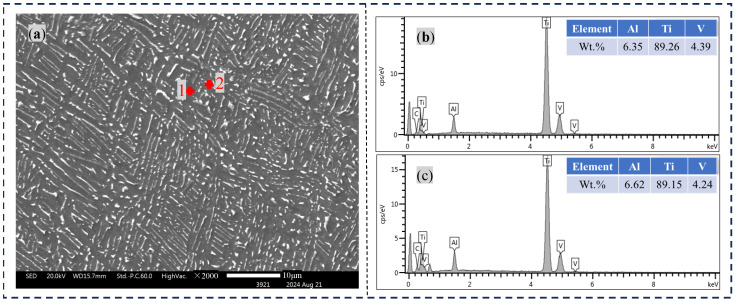
EDS results in the raw material without DCT. (**a**) Scanning electron microscopy morphology, (**b**) Element content of point 1, (**c**) Element content of point 2.

**Figure 5:**
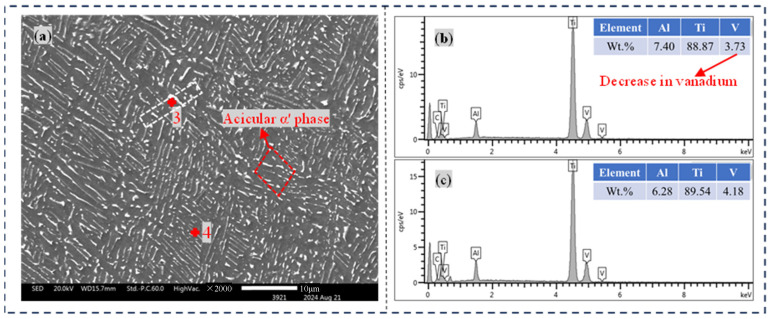
EDS results in the base material treated with 18 h of DCT. (**a**) Scanning electron microscopy morphology, (**b**) Element content of point1, (**c**) Element content of point.

Figure 4 and Figure 5 provide a visual representation of the characteristic microstructure and the elemental content in the TC4 Ti alloy with and without DCT. Figure 4a shows the SEM morphology and point scan location of the base material area without DCT. The peak intensity of the energy spectrum at the two scan points is shown in Figure 4b,c, which includes the element mass fraction of the characteristic points. The points where spectra 1 are located are the darker α phase, composed of Ti, Al, and V. The points where spectra 2 are located are the clearly bright β phases, which are composed of Ti and Al elements, and the elemental content is almost the same as that of spectra 1.

Figure 5 shows the SEM morphology and point scanning position of the base material with 18 h of DCT. The peak intensity of the energy spectrum at the two scan points is shown in Figure 5b,c, which also includes the element mass fraction of the characteristic points. The point where spectrum 3 is located is the α′ phase, and its alloy elements are consistent with spectrum 4, but its content of the element V is 0.5% lower than that of the β phase. From the above research, it was found that the β phase in the TC4 titanium alloy base material transforms into a needle-like martensite α′ phase with DCT. The solid solubility of the α phase relative to the element V decreases. Therefore, during DCT, an amount of the element V precipitates, and β has an infinite solid solubility relative to V. Meanwhile, the β phase that has not undergone phase transformation becomes the destination of the element V with DCT. However, due to the low temperature and the low atomic diffusion rate in the DCT state, the content of V in the β phase is increased. But the improvement is not significant.

#### 3.2.4. EBSD Analysis

**Figure 6:**
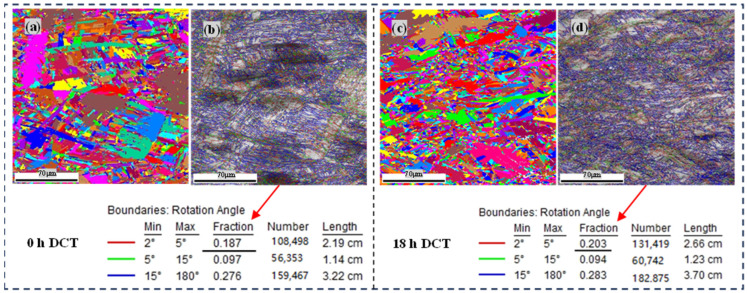
EBSD orientation maps of base material with and without DCT. (**a**) IPF—0 h, (**b**) Grain boundary diagram—0 h, (**c**) IPF—18 h, (**d**) Grain boundary diagram—18 h.

As shown in Figure 6, an EBSD orientation map of the test area was obtained in the untreated original state and after 18 h of DCT. As shown in Figure 6a, the proportion of small angle grain boundaries in the original state was 18.7%, but the proportion of small angle grain boundaries in the test area increased to 20.3% with 18 h of DCT, as shown in Figure 6a, indicating that the proportion of small angle grain boundaries significantly increased with 18 h of DCT. The reason for the increase in the proportion of small angle grain boundaries may be attributed to the increases in the dislocation density inside the base material, causing entanglement and rearrangement of the dislocations. As is known, the energy of small angle grain boundaries is low, and the bonding ability of grain boundaries is stronger. Therefore, an increase in the proportion of small angle grain boundaries has a positive effect on improvement in the material’s plasticity and toughness.

**Figure 7:**
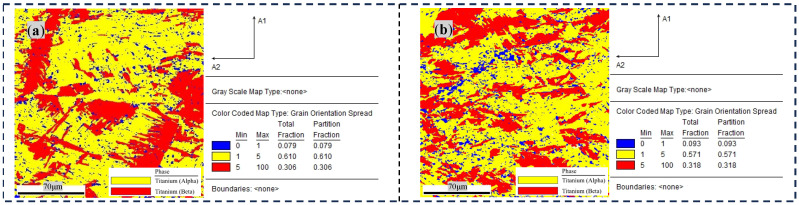
Phase characteristics of base material with and without DCT: (**a**) 0 h and (**b**) 18 h.

The first aspect is linked to the grain size in the whole view displayed by Figure 7. By comparing Figure 7a,b, we can see the grain size of the sample treated with 18 h of DCT decreases in comparison to that in the untreated sample, which indicates that DCT can refine the grain size. The second aspect about the kinds of different grains with and without DCT is the statistical crystal characteristics in the samples. The blue, yellow, and red colors represent recrystallized, sub-grains, and deformed crystal grains, respectively. In the untreated sample, most of the crystal grains are deformed because the original alloy has been subjected to the rolling process. Meanwhile, the number of deformed grains decreased obviously and the number of recrystallized grains and sub-grains increased with 18 h of DCT. Cold contraction stress induces a large amount of dislocation. Dislocation cells, due to tangled dislocation, accelerate the generation of more vesicular sub-structures, as sub-grains. The dislocations at the grain boundary possess a higher energy, which will be absorbed by the grain boundary of the sub-grains. This is beneficial in decreasing the deformation energy in materials, during which process the sub-grain boundaries will transform from small-angle grain boundaries into large-angle ones and form dynamic recrystallized grains.

**Figure 8:**
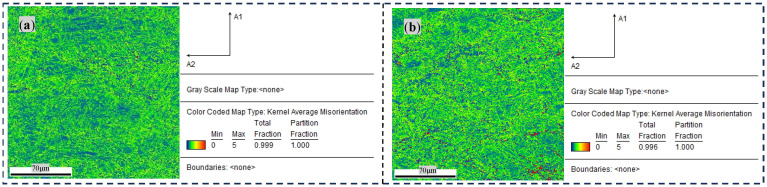
KAM maps of base material with and without DCT: (**a**) 0 h and (**b**) 18 h.

Figure 8 shows the distribution of the KAM (kernel average misorientation) in the untreated original state and when treated with 18 h of DCT. As described, the proportion of green areas in the test area increased, indicating that dislocations accumulated after 18 h of DCT and the dislocation density increased.

#### 3.2.5. TEM Analysis

**Figure 9:**
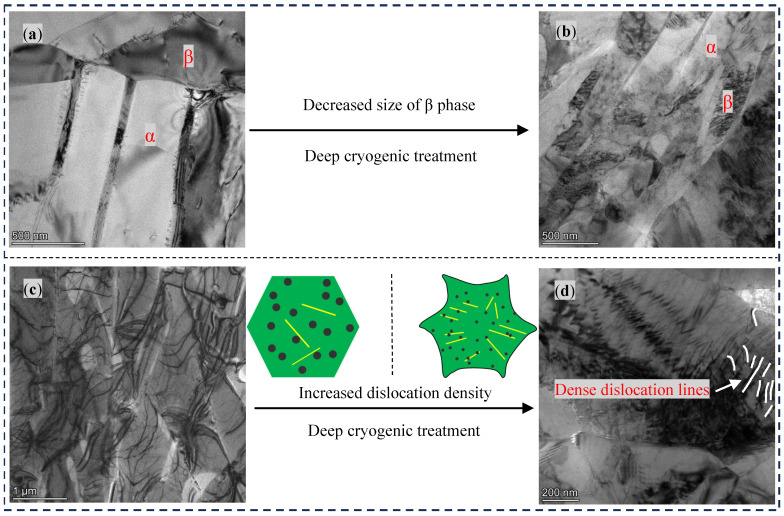
Transmission electron microscopy morphology of base material with and without DCT. (**a**,**c**): 0 h; (**b**,**d**): 18 h.

Detailed detection of the microstructures was conducted using TEM, as shown in Figure 9a,b. It was found that the size of some of the β phase particles reduces after 18 h of DCT.

Moreover, the dislocation density of the sample significantly increased with 18 h of DCT, as shown in Figure 9c,d. The reason for this is that volume shrinkage and lattice distortion intensify the accumulation of deformation energy inside the material with DCT, which can generate a large number of dislocations and sub-grain structures, leading to instability of the crystals. After the sample is taken out of a cryogenic environment, the interior of the sample spontaneously returns to a low-energy state through recovery, leading to a phase transition.

### 3.3. Comparative Analysis of Mechanical Properties

#### 3.3.1. Comparison of Hardness

Hardness characterizes the ability of a material to resist local pressure from hard objects on its surface and is one of the important indicators for characterizing the mechanical properties of materials. The average hardness of the TC4 titanium alloy base material used in this experiment is 331.2 HV_0.5_.

**Figure 10:**
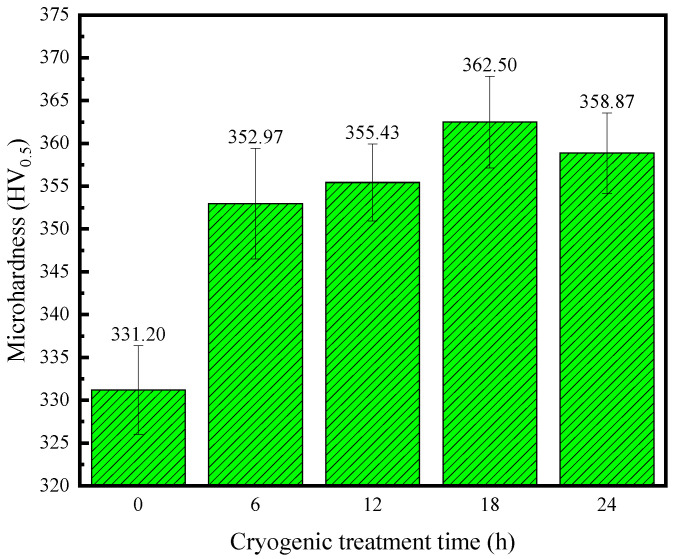
Microhardness of the base metal treated with different DCT times.

Figure 10 shows the average hardness of the base material treated with different DCT times. It can be observed from Figure 9 that the hardness of the TC4 titanium alloy is noticeably enhanced upon the application of DCT. With an increase in the DCT time, the improvement in hardness becomes more obvious. The microhardness reaches the maximum value of 362.5 HV_0.5_ with 18 h of DCT, which is about 9.4% higher than that of the TC4 titanium alloy base material without DCT. However, the hardness decreases to 358.89 HV_0.5_ when the DCT time increases to 24 h but is still much higher than that of the base material without DCT.

The reasons why the microhardness of the TC4 titanium alloy base material improved with DCT are mainly the following. Firstly, phase transformation occurred with DCT, the β phase transformed into the α′ needle-like martensite phase, and the relative surface hardness of α′ needle-like martensite played a strengthening role. Secondly, the internal stress generated by DCT inside the TC4 titanium alloy caused an improvement in dislocation density, leading to a corresponding increase in the interaction between dislocations and resulting in a hardening effect. The third reason is that DCT has a refining effect on the grain size. An increase in grain size means an increase in grain boundaries, which can hinder the movement of dislocations and increase the deformation resistance of a material. These macroscopic mechanical properties manifest as an improvement in hardness. However, when the DCT reaches 24 h, there is a certain degree of decrease in the hardness. This phenomenon can be attributed to the refined grains becoming uniform and reaching a relatively balanced state under the action of internal stress with an increase in DCT time, which consequently results in a slight decrease in hardness.

#### 3.3.2. Comparison of Tensile Properties

**Figure 11:**
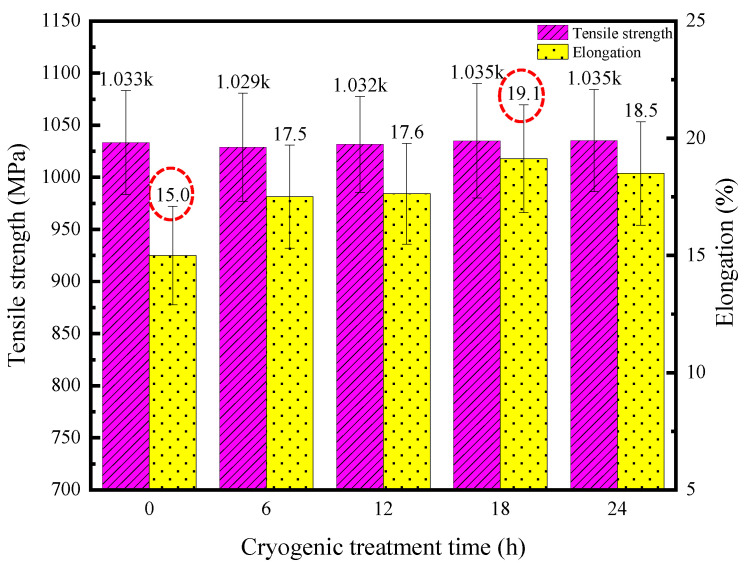
Tensile properties of the base metal with different DCT times.

As illustrated in Figure 11, it can be observed that there is little change in the tensile strength with and without DCT, fluctuating around 1033.0 MPa, while the elongation exhibited a notable improvement. As can be seen, the elongation increases significantly with 6 h of DCT compared to without DCT, which reaches 17.5%. Furthermore, the elongation exhibited a substantial augmentation of 27.3% with 18 h of DCT, rising from 15.0% to 19.1%, compared to without DCT. Meanwhile, the elongation decreases to some extent with 24 h of DCT. The reasons for the improvement in elongation with DCT are as follows: (1) DCT causes a transformation from the β phase into the α′ needle-like martensite phase. Due to the dense hexagonal structure of the α′ phase and the body-centered cubic structure of the β phase, the density of the α′ phase is much higher than that of the β phase. Moreover, with an extension in the DCT time, the internal α′ needle-like martensite will grow to a certain extent, resulting in a certain degree of improvement in the material’s toughness. (2) The vacancies inside the TC4 titanium alloy base material decrease due to volume shrinkage with DCT, thereby increasing the density of the material and improving its elongation after fracture. A combination of these two reasons leads to a certain increase in the elongation after fracture of the TC4 titanium alloy base material.

As is known, the grains inside the TC4 titanium alloy show a certain degree of refinement with DCT. In theory, the tensile strength of the TC4 titanium alloy base material should be improved to some extent by DCT, but the variation is not significant. The main reasons for this are as follows: (1). “Microstructure stability”: Although the TC4 titanium alloy base material undergoes a transformation from the β phase into the α′ needle-like martensite phase with DCT (deep cryogenic treatment), its microstructure remains relatively stable and only undergoes minor changes. (2). “High tensile strength”: TC4 titanium alloy already possesses a very high tensile strength. Consequently, the subtle changes in its internal microstructure due to DCT do not significantly impact its tensile strength.

**Figure 12:**
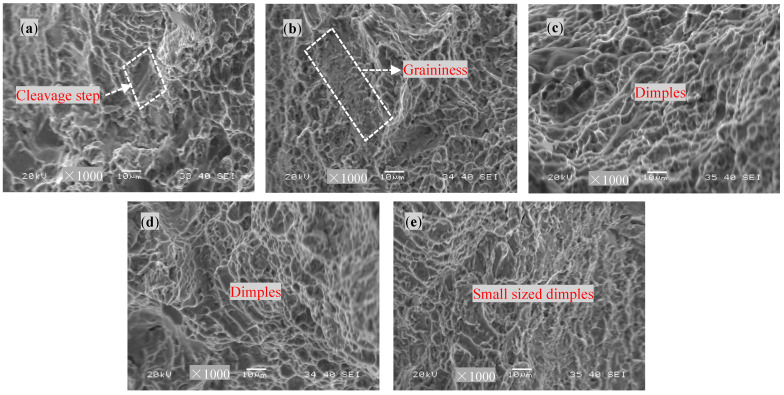
The tensile fracture morphologies of the base metal with different DCT times: (**a**) 0 h, (**b**) 6 h, (**c**) 12 h, (**d**) 18 h, and (**e**) 24 h.

Figure 12 shows the tensile fracture morphologies of the base material treated with different DCT times. The fracture surface of the raw base material without DCT exhibits the presence of cleavage steps, indicating a combination of brittleness and ductility, with a dominant ductility component. Furthermore, the fracture surface is composed of small dimples after 6 h of DCT, and the fracture areas exhibit a noticeable particle morphology. When the DCT time increases to 12 h and 18 h, the dimples on the fracture surface become larger and dense, and the dimples on the fracture surface are significantly deeper with 18 h of DCT, signifying an improvement in the plasticity, which shows the characteristic of ductile fracture. When the DCT time increases to 24 h, the fracture surface is still composed of a large number of dimples, but the size and depth of the dimples decrease.

According to the testing results, it can be concluded that the tensile properties are improved to a certain extent with DCT. This is because considerable volumetric thermal shrinkage occurs inside the material at such a low temperature (−196 °C), and this shrinkage also causes compression of the lattice structures at the molecular level. This compression effect is observed at the lattice structure level; this phenomenon occurs more dominantly in the β phase, which has a BCC lattice structure, rather than the α phase, with an HCP lattice structure, where molecular movements are more difficult [23].

#### 3.3.3. Comparison of Impact Properties

**Figure 13:**
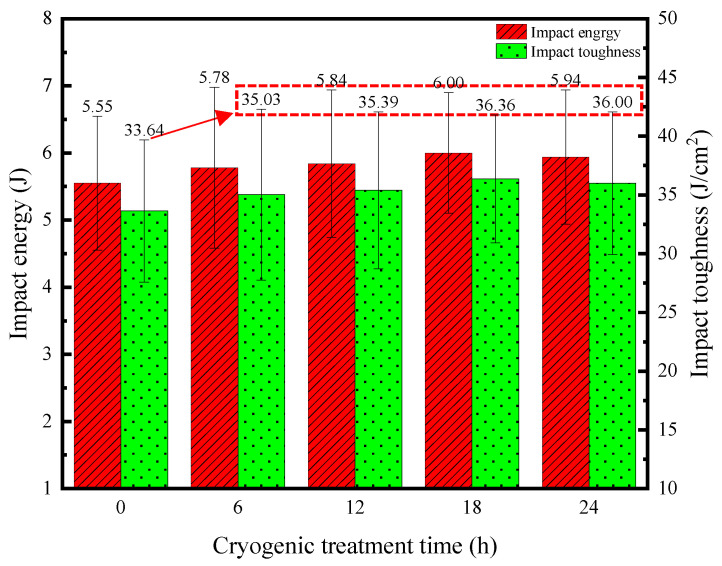
Impact properties of the base metal with different DCT times.

Figure 13 shows the impact test results for the base material with different DCT times. It can be observed that the application of 18 h of DCT resulted in a notable enhancement in the impact toughness, showing an 8.09% increase from 33.64 J/cm^2^ to 36.36 J/cm^2^ compared to that in the sample without DCT. The trend in changes in toughness is consistent with that for elongation after fracture which was discussed in the previous section, and both of these indicators are used to characterize the plastic toughness index of the material. The reasons for the improvement in impact toughness after a certain period of DCT mainly cover two aspects. One reason is the refinement of the internal structure of the TC4 titanium alloy by DCT. The second reason is the increase in density caused by phase transition and the decrease in the internal free volume of the base material.

However, when the DCT time was increased to 24 h, the mechanical performance indicators of the TC4 titanium alloy base material decreased to a certain extent. This observation is consistent with the findings discussed in relation to the tensile properties. Therefore, when DCT is carried out on TC4 titanium alloy base materials, an appropriate DCT time should be selected. A prolonged DCT time cannot improve the mechanical properties of the material further.

**Figure 14:**
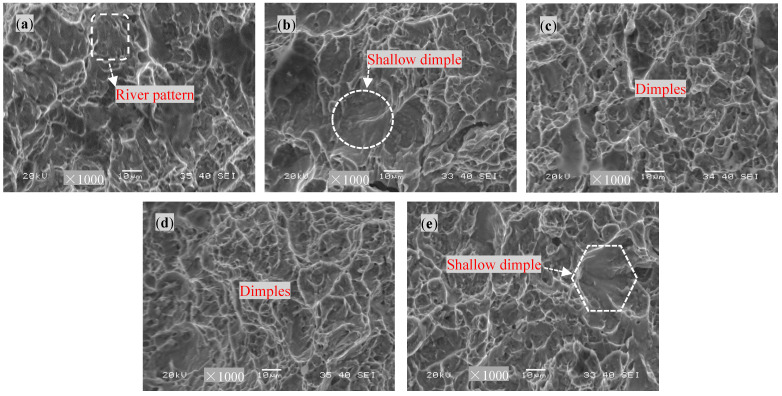
The impact fracture morphologies of the base metal treated with different DCT times: (**a**) 0 h, (**b**) 6 h, (**c**) 12 h, (**d**) 18 h, and (**e**) 24 h.

Figure 14 shows the impact fracture morphologies of the base metal treated with different DCT times. For the sample without DCT, the impact fracture exhibits the presence of a river pattern, and the depth of the dimples becomes shallow when the DCT time reaches 6 h. When the DCT time was increased to 12 h, a number of dimples could clearly be observed, significantly more than on the impact surface of the sample treated for 6 h. When the DCT time was increased to 18 h, a large number of uniform and dense dimples were found at the fracture, and the depth of the dimples was significantly increased compared to the sample treated with 12 h. Nevertheless, when the DCT time reached 24 h, concentration of stress tended to occur more readily, and the size of the dimples at the fracture surface was greatly improved, but the depth and quantity of the dimples decreased, which is counterproductive for impact performance [24].

## 4. Conclusions

(1) DCT has a significant effect on the grain refinement of the TC4 titanium alloy. Obvious grain refinement behavior can be observed with 6 h of DCT, and the grain refinement inside the TC4 titanium alloy base material becomes more obvious with an increase in the DCT time. In addition, DCT promotes the transformation of the β phase into the α′ phase in the TC4 titanium alloy base material. Through SEM point scanning of the element V, it was found that the β phase inside the base material had transformed into the α′ phase.
(2) The hardness of the TC4 titanium alloy base material was significantly improved with DCT. In particular, the hardness of the base material increased from 331.2 HV_0.5_ to 362.5 HV_0.5_ with 18 h of DCT, presenting a 9.5% improvement compared to the sample without DCT.
(3) The sample subjected to 18 h of DCT experienced little effect on its tensile strength, but there were notable improvements in elongation (19.13%) and toughness (36.36 J/cm^2^), demonstrating increases of 27.53% and 8.09%, respectively, compared to the sample without DCT. Additionally, the tensile and impact fracture morphologies displayed characteristics consistent with ductile fracture.

## Figures and Tables

**Table 1:** Chemical composition of TC4 titanium alloy.

Element	Al	V	Fe	C	N	H	O	Ti
Content (wt.%)	5.970	3.930	0.086	0.007	0.006	0.012	0.150	Balance

**Table 2:** Mechanical properties of TC4 titanium alloy.

Mechanical Properties	Tensile Strength	Yield Strength	Elongation	Impact Toughness	Hardness
Value	1033.3 MPa	1000.0 MPa	15.0%	33.6 J/cm^2^	331.2 HV

## Data Availability

The original contributions presented in the study are included in the article, further inquiries can be directed to the corresponding author.
